# Byline or Botline? The Dilemma of Artificial Intelligence in Medical Scholarship

**DOI:** 10.1016/j.mcpdig.2025.100259

**Published:** 2025-08-28

**Authors:** James Connor

**Affiliations:** Department of Urology, Mater Misericordiae University Hospital, Royal College of Surgeons in Ireland, Dublin, Ireland

As a newly minted physician stepping onto the academic stage, the need to publish has become more than a professional milestone—it is a mandate. Advancement, fellowships, and academic recognition are all deeply intertwined with the metrics of publication. The pressure to generate research output in the earliest years of medical practice is immense, even while navigating grueling clinical schedules and the steep learning curve of operative independence. Into this high-stakes environment enters artificial intelligence (AI), with a suite of tools that promises to accelerate writing, synthesize literature, and streamline the often arduous process of academic authorship. For many, particularly those at the beginning of their academic careers, the appeal is immediate, and the temptation is difficult to resist.

Large language models (LLM) like ChatGPT, Elicit, and SciSpace are no longer fringe novelties. They are, in many circles, the default starting point for drafting abstracts, structuring manuscripts, or translating clinical observations into academic prose. Their capabilities are impressive. In minutes, a generative AI tool can produce a coherent introduction and offer a literature summary while simulating the tone of high-impact journals. For international trainees or doctors for whom English is not a first language, these platforms offer an opportunity to compete in an academic landscape that has historically biased authors from particular parts of the world or within certain circles. By inputting a few short prompts, these tools can generate convincing journal articles, complete with statistics and commentary that have the potential to make it through a peer review selection process that we as academics hold so dear.

For all their power, these tools pose profound questions to the future of academic medicine. The tension between technological assistance and intellectual authenticity has never been more pressing. As young doctors, we must ask ourselves: are we sharpening our academic voices, or outsourcing them entirely? Are we generating scholarship or content?

There is a seductive ease to the use of AI in manuscript preparation. With a few prompts and keywords, a scaffold appears; with minor edits, it can seem publication-ready. But this convenience can mask a dangerous erosion of rigor. Generative models, after all, do not understand the scientific method. They do not grasp medical nuance, the gravity of a clinical misstep, or the slow accumulation of evidence over time. They are trained on what has been written, not necessarily on what is true. An AI model can produce a fluent paragraph about laparoscopic outcomes in hernia repair, but it cannot judge whether the cited data is methodologically sound or even whether the sources exist. Too often, it cannot distinguish between landmark trials and speculative editorials.

The implications are troubling. Junior researchers, eager to publish, may accept AI-generated output at face value. They may trust references that are fabricated or conclusions that are logically inconsistent. They may unknowingly submit manuscripts that, while polished in appearance, are hollow in substance. This risk is not hypothetical. Many editors have already noted a rise in submissions that read like simulations of academic work—grammatical and organized, but devoid of critical thought or original insight.^1^

In parallel, we are witnessing a proliferation of low-tier and predatory journals that thrive in this ecosystem. These journals, often with minimal peer review and aggressive publication fees, cater explicitly to early-career physicians under pressure to publish. The synergy is clear: AI makes it easier to produce content, and predatory journals offer an outlet with little scrutiny. Together, they threaten to dilute the quality of medical literature and to mislead readers, clinicians, and policymakers alike.

Another emerging concern is the erosion of critical self-assessment. As AI simplifies the process of data analysis and prose generation, there’s a risk that rigorous interpretation gives way to convenience. There is growing evidence that reliance on AI language tools can impair users’ ability to critically analyze prose.^2^ The result is a proliferation of publications, where, given enough searching, evidence can be found to support virtually any claim. This ease may dilute academic standards and hinder genuine progress in the profession.

A final concern lies in the erosion of the traditional understanding of authorship—the belief that scholarly work reflects substantial intellectual effort, rigorous revision, and personal investment. Historically, each publication has been perceived as the product of sustained inquiry and reflection, lending weight to its conclusions and trust in its authors. The introduction of generative AI disrupts this assumption. As machines increasingly contribute to manuscript drafting, readers may begin to question the authenticity, depth, and human judgment behind published prose. Indeed, this piece was neither written, edited, nor revised by generative AI or LLM, but would you know if it was? And if so, would you think it truly mattered?

This is not a call to reject AI outright. To do so would be both impractical and short-sighted. These tools, when used ethically and transparently, can indeed augment our productivity. They can help us communicate more clearly, organize our ideas more efficiently, and overcome the inertia of the blank page. But they are no substitute for the essential work of research: asking the right questions, designing thoughtful studies, interpreting data within the context of clinical practice, and engaging with the literature critically. As AI becomes increasingly embedded in academic workflows, all stakeholders involved in medical publishing have a role to play in defining its appropriate and ethical use.

How should publishing systems deal with the rising use of AI and LLM? While algorithmic tools exist to detect AI-generated content, their accuracy remains inconsistent. More fundamentally, we must ask, is it the role of journals to police the presence of AI in prose or to reject submissions on principle simply because such tools may have been used? Given the near ubiquity of AI in the research ecosystem, this approach seems reductive. Firstly, the onus should lie with the authors themselves to declare use of LLM in the generation of manuscripts and detail exactly what it was used for. This should not be met with scrutiny but rather treated as an extension of the trust-based model we already apply to disclosures of conflicts of interest and ethical conduct in research. Second, the larger task may increasingly fall on peer reviewers and editorial boards to shift their focus—not merely evaluating fluency or stylistic polish, which AI can now easily simulate, but assessing the depth of insight, originality of thought, and integrity of scientific contribution.

For trainees, the path forward must begin with education. We need explicit guidance on how and when to use AI tools in academic writing. Medical schools and training programs must begin to incorporate discussions on AI literacy—not just technical skills, but ethical frameworks. Mentors should model transparency, disclosing their own use of AI and reinforcing standards for originality and attribution.

It is also time to reconsider how we assess academic productivity in the early stages of a medical career. Publication volume, while easy to measure, is a poor proxy for curiosity, insight, or clinical acumen. We should reward the quality of thought, the courage to tackle difficult questions, and the integrity of the methods more than the frequency of bylines. In doing so, we reduce the incentive to cut corners or chase quantity at the expense of substance.

As a new doctor in a hypercompetitive academic landscape, I understand the temptation to lean on these tools more heavily than we should. But we must remember that our writing, like our practice, must be accountable. Just as we would never delegate a complex case to an untrained assistant without supervision, we should not delegate our scholarly voice to algorithms without rigorous oversight. Our responsibility is not only to our own careers but to the body of medical knowledge that will inform and shape care for generations to come.

LLMs have advanced rapidly and so quickly become so deeply ingrained in the fabric of medical research that it is counterproductive to regard them as an enemy. The AI, when approached with clarity and conscience, can become more than just a shortcut—it can be a catalyst. It can prompt us to ask better questions, distil complex insights, and communicate with greater clarity across borders and disciplines. Rather than replacing our intellectual work, it can sharpen it, challenging us to define what truly constitutes thought, authorship, and originality in a digital age. If we embrace these tools with transparency and rigor, we have the chance not only to become faster researchers or more efficient writers but better scientists: more reflective, more curious, and more connected to the evolving landscape of knowledge that underpins our care.

## Potential Competing Interests

The authors report no competing interests.
